# Does dietary fat affect inflammatory markers in overweight and obese individuals?—a review of randomized controlled trials from 2010 to 2016

**DOI:** 10.1186/s12263-017-0580-4

**Published:** 2017-10-04

**Authors:** Vibeke H. Telle-Hansen, Jacob J. Christensen, Stine M. Ulven, Kirsten B. Holven

**Affiliations:** 1 Faculty of Health Sciences, Oslo and Akershus University College of Applied Sciences, Postbox 4, St. Olavsplass, 0130 Oslo, Norway; 2 Department of Nutrition, Institute of Basic Medical Sciences, Faculty of Medicine, University of Oslo, Postbox 1046, Blindern, 0317 Oslo, Norway; 3 The Lipid Clinic, Oslo University Hospital Rikshospitalet, P.P. box 4950, Nydalen, 0424 Oslo, Norway; 4 Norwegian National Advisory Unit on Familial Hypercholesterolemia, Oslo University Hospital Rikshospitalet, P.O. box 4950, Nydalen, 0424 Oslo, Norway

**Keywords:** Overweight, Obese, Inflammation, Metabolic, Dietary fat, Fatty acids, CRP, IL-6, TNFα, RCT

## Abstract

**Background:**

Obesity, a major cause of death and disability, is increasing worldwide. Obesity is characterized by a chronic, low-grade inflammatory state which is suggested to play a critical role in the development of obesity-related diseases like cardiovascular diseases and type 2 diabetes. In fact, in the hours following consumption of a meal, a transient increase in inflammatory markers occurs, a response that is exaggerated in obese subjects. Dietary composition, including content of dietary fatty acids, may affect this inflammatory response both acutely and chronically, and thereby be predictive of progression of disease. The aim of the review was to summarize the literature from 2010 to 2016 regarding the effects of dietary fat intake on levels of inflammatory markers in overweight and obesity in human randomized controlled trials.

**Methods and results:**

We performed a literature search in MEDLINE, EMBASE, and PubMed databases. The literature search included human randomized controlled trials, both postprandial and long-term interventions, from January 2010 to September 2016. In total, 37 articles were included. Interventions with dairy products, vegetable oils, or nuts showed minor effects on inflammatory markers. The most consistent inflammatory-mediating effects were found in intervention with whole diets, which suggests that many components of the diet reduce inflammation synergistically. Furthermore, interventions with weight reduction and different fatty acids did not clearly show beneficial effects on inflammatory markers.

**Conclusion:**

Most interventions showed either no or minor effects of dietary fat intake on inflammatory markers in overweight and obese subjects. To progress our understanding on how diet and dietary components affect our health, mechanistic studies are required. Hence, future studies should include whole diets and characterization of obese phenotypes at a molecular level, including omics data and gut microbiota.

## Background

### Obesity and inflammation

The prevalence of obesity is increasing worldwide. The number of affected individuals is nearly doubled between 1980 and 2008 [1], and estimates show that by 2030, prevalence will increase by 65 million in the USA and 11 million in the UK [2]. Obesity, defined as a body mass index (BMI) of 30 kg/m^2^ or higher, is independently associated with increased mortality and is an important risk factor for metabolic diseases, such as cardiovascular diseases (CVD) and type 2 diabetes (T2D) [3]. Obesity can be considered a consequence of prolonged imbalance between energy intake and expenditure, driven by a complex interplay between genes, diet, and other environmental factors [4, 5]. Interestingly, the chronic low-grade inflammatory state of obesity [5–7] is suggested to play a critical role in the development of obesity-related metabolic dysfunction [8]. Adipose tissue contains adipocytes and infiltrated macrophages, both of which release a spectrum of similar inflammatory mediators, including acute-phase proteins (like PAI-1), cytokines (like IL-6, TNFα), and chemokines (like MCP1). Consequently, circulating levels of inflammatory markers are elevated in human obese subjects and associate with obesity-related parameters [9–12].

### Dietary fat and inflammation

Lifestyle factors, such as diet and exercise, play an important role in the development and progression of obesity and its comorbidities. Specific dietary factors, such as dietary fat, may modulate inflammation and thereby risk of disease in humans [13]. Dietary fat is composed of different fatty acids, like saturated fatty acids (SFA) and *trans* fatty acids, monounsaturated fatty acids (MUFA), polyunsaturated fatty acids (PUFA) of both omega (n) 6- and n3-family, and conjugated linoleic acid (CLA). The inflammation-specific modulatory effect of dietary fat may for example act via the eicosanoid metabolism or as regulators of membrane and cytosolic signaling through activation of gene expression. Dietary fat can also directly regulate gene expression by acting as ligand for transcription factors, such as the peroxisome proliferator-activated receptors (PPAR) and liver X receptors (LXR). Importantly, the inflammatory response differs depending on the type of fatty acid. Generally, while SFA and *trans* fat are considered pro-inflammatory, the PUFA and especially the long-chain (LC) n3 fatty acids are considered anti-inflammatory. Being a precursor of pro-inflammatory eicosanoids, n6 PUFA have been suggested to mediate pro-inflammatory effects and thereby increase the risk of chronic diseases in humans [14]. However, despite the general acceptance that n6 PUFA are pro-inflammatory, several studies show that humans with the highest intake or plasma level of n6 PUFA have the lowest inflammatory status and hence do not support a pro-inflammatory effect [15].

In the hours following the consumption of a meal, a transient increase in circulating inflammatory markers occurs [16], which potentially contributes to endothelial dysfunction and vascular disease [17]. The post-prandial inflammatory reaction appears to be triggered mainly by triglycerides and SFA, in addition to total energy and glucose content of the meal [16, 18]. Interestingly, this post-prandial inflammatory response is exaggerated in obese subjects [8, 19]. Persistent increased post-prandial exposure produces a state of chronic low-grade inflammation, characterized by increased systemic levels of pro-inflammatory cytokines (TNFα, IL-1β, and IL-6) and chemokines [17], which is a critical player in the development of many lifestyle diseases.

Research on diet-related health effects has traditionally examined single nutrients. Although successful, this approach has largely changed towards the examination of food, diets, or dietary patterns. Humans do not eat single nutrients; they eat meals with complex mixtures of different nutrients. In addition, many nutrients have synergistic or interactive effects. Previous studies have shown that healthy dietary patterns, characterized by increased PUFA intake in place of SFA, are associated with decreased chronic low-grade inflammation, in particular decreased level of TNFα and IL-6 [8].

Obesity-related inflammation is mainly mediated by the increased fat mass in the obese state; however, it may be modulated by chronic or acute exposure to dietary fat. Calder and coworkers performed a comprehensive review of dietary factors and inflammation in 2011, including both dietary patterns and dietary components (whole grains, fruits, vegetables, nuts, soya, coffee, tea, cocoa, fiber, milk peptides, vitamin E, vitamin C, fatty acids, carbohydrates, iron, vitamin D, phytochemicals, gut microbiota, prebiotics, and probiotics) [8]. They conclude that a healthy dietary pattern, like the Mediterranean diet, is associated with decreased low-grade inflammation in both healthy and obese individuals. They further conclude that important protective factors in the diet are whole grains, fiber, fruits, vegetables, fish, PUFA, and especially n-3 PUFA, vitamin C, vitamin E, and carotenoids. Dietary factors that are inconclusive or have no effect on inflammation include nuts, tea, coffee, cocoa, flavonoids, alcohol, milk peptides, vitamin D, probiotics, and prebiotics, while oxidized lipids, SFA, and *trans* fatty acids promote inflammation [8]. The aim of the present review was to summarize the latest research findings (2010–2016) in the area of dietary fat and inflammatory markers in overweight and obesity in human randomized controlled trials.

## Method

**Fig. 1:**
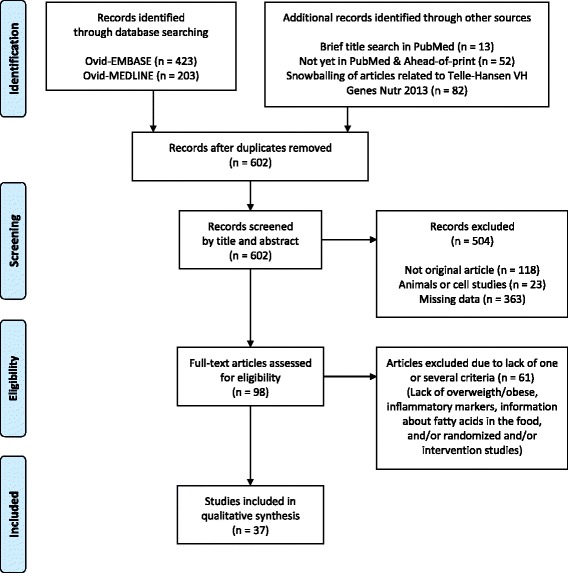
Flow chart of the study selection

To identify relevant studies, we performed a literature search in MEDLINE, EMBASE, and PubMed. The search was performed in September 2016 and was limited to publications from January 2010 to September 2016. Only original articles and randomized intervention trials in overweight and obese humans were included. Furthermore, only studies with information about intake of total fat, SFA, MUFA, and PUFA, or with total fatty acid profile in the foods or whole diet were included. Inflammatory markers included in this article were defined as pro-inflammatory cytokines, acute-phase proteins, and adhesion molecules and chemokines (CRP, TNFα, IL-6, ICAM, VCAM, and MCP1). In addition, altered proteome and mRNA transcripts of such markers were included. We included studies which clearly or possibly fulfilled the following criteria: overweight/obese subjects, intervention with fatty acids, and at least one inflammatory marker measured. Moreover, we excluded studies that clearly fulfilled at least one of the following criteria: not original study (for example editorial, review or conference paper), animal study, or lack of inclusion criteria measurements (as defined previously). Duplicate articles were removed. In total, 37 articles were reviewed in full text and included in the present article. Figure 1 shows the flow chart of the study selection.

## Results and discussion

### Dietary fat and inflammatory markers

In the present review, we discuss the effects of dietary fatty acid intake on markers of inflammation in overweight and obese subjects, as documented by post-prandial and short- and long-term intervention trials (parallel and cross-over design; 3 weeks to 1 year) (Tables 1 and 2).

**Table 1:** Randomized controlled trials and inflammatory response

Parallel, cross-over, and post-prandial studies											
Study	Subjects	*N* (sex)	Age (years)	Duration and study design	Intervention/intake	hsCRP/CRP	TNFα	IL-6	ICAM	VCAM	MCP1
Dairy											
Van Meijl & Mensink, 2010, The Netherlands	Healthy BMI 32.0	35 (M/F)	49.5	8 weeks cross-over	Dairy products: 500 mL low-fat (1.5%) milk and 150 g low-fat (1.5%) yogurt per day Control products: 600 mL fruit juice and 43 g fruit biscuits (3 pieces) per day		↔	↔	↔	↔	↔
Van Meijl & Mensink, 2011, The Netherlands	Healthy BMI > 27	35 (M/F)	M: 52.3 F: 47.2	8 weeks cross-over	Dairy products: 500 mL low-fat (1.5%) milk and 150 g low-fat (1.5%) yogurt per day Control products: 600 mL fruit and 43 g fruit biscuits (3 pieces) per day	↔					
Nestel et al*.*, 2013, Australia	Healthy BMI 30.1	12 (M/F)	60.5	3 weeks cross-over	Full-fat diet (fermented): Cheddar cheese (85 g/day) and full-cream yogurt (600 g/day). Full-fat diet (non-fermented): butter (30 g/day) and cream (70 mL/day). Low-fat diet: 1% fat milk (400 mL/day) and < 1% fat yogurt (200 g/day)	↔	↔	↑with full-fat non-fermented diet vs. full-fat fermented and low-fat diet	↔	↔	↔
Werner et al., 2013, Denmark	Healthy BMI < 35	38 (M/F)	50–70	12 weeks parallel	All buns contained 13 g of butter, which yielded a fat intake of from the test diet of 39 g/day Grazing: buns with butter from pasture-grazing cows Conventional: buns with butter from conventional fed Danish cows	↔					
Druin-Chartier et al., 2015, Canada	Abdominal obesity BMI 31.9	27 (F)	57	6 weeks cross-over	Isocaloric milk drinks; 20% of calories from partly skimmed milk (2% fat) and control NCEP milk-free diet. MILK phase: 3.2 serv/day. NCEP control phase: 0 serv/day.	↔			↔	↔	
Venkatramanam et al*.*, 2016, Canada	Healthy BMI 25–30	15 (M/F)	46.6	8 weeks cross-over	1 L/day. Group 1: milk natural enriched with CLA (NCLA) (4.2%) containing [cis 9, trans 11 (c9,t11)] providing 1.3 g CLA/day. Group 2: milk enriched with synthetic CLA (SCLA) (4.2%) [trans 10, cis 12 (t10, c12) and c9, t11] providing 1.3 g CLA/day. Group 3: Untreated milk providing 0.2 g CLA/day	↔	↔				
Nuts											
Lopez-Uriarte et al., 2010, Spain	MetS BMI 25–35	50 (M/F)	51.8	12 weeks parallel	Nut group: AHA dietary guideline enriched with a daily supplement of 30 g/day of mixed raw nuts with skin (15 g walnuts, 7.5 g almonds and 7.5 g/day hazelnuts) Control group: quantitative recommendations according to the AHA dietary guidelines				↔	↔	
Casas-Agustench et al., 2011, Spain	MetS Nut gr: BMI 31.6 Control: BMI 30.0	50 (M/F)	18–65	12 weeks parallel	Nut diet group: prudent diet supplemented with nuts; daily supplement of 30 g of raw unpeeled nuts (15 g of walnuts, 7.5 g of almonds and 7.5 g of hazelnuts) Control diet group: prudent diet	↔		↓ with nuts vs. control (disappear when weight adjusted)			↔
Bakthtiary et al., 2012, Iran	MetS BMI 27.5–28.8	75 (F)	60–70	12 week parallel	Group A: 35 g soy nuts Group B: 35 g TSP (Textured soy protein) Group C: control	↔					
Ling Tey et al., 2013, New Zealand	Healthy BMI 30.6	107 (M/F)	42.5	12 weeks parallel	Group 1: 30 g/day of hazelnuts Group 2: 60 g/day of hazelnuts Control: no nuts	↔		↔	↔	↔	
Barbour et al., 2015, Australia	Healthy BMI 31	61 (M/F)	65	12 weeks cross-over	Peanut diet: 84 g (M) or 56 g (F) of high oleic peanuts 6 days per week Control diet: habitual diet devoid of nuts	↔					
Vegetable oils											
Gagliardi et al., 2010, Brazil	MetS BMI 31.3	53 (M/F)	47.4	5 weeks parallel	Butter: 18 g/day No-trans margarine: 36 g/day Plant sterol margarine: 30 g/day and 2.4 g plant sterol/day Equal amounts of total lipids (12.6–12.86 g/day) and calories (96–115.5/day) in the three groups	↔		↔			
Masson & Mensink, 2011, The Netherlands	Healthy BMI 25–30	14 (M)	18–70	0–480 min post-prandial Cross-over	2 muffins and a glass of water (250 mL). Comparable energy content (4095 kJ and 4253 kJ, respectively) Butter meal: 50 g butterfat Margarine meal: 40 g margarine plus 10 g safflower oil		↓ with margarine vs. butter	↓ with margarine vs. butter	↔	↓ with margarine vs. butter	↔
Bjermo et al., 2012, Sweden, HEPFAT trial	Abdominal obese PUFA diet: BMI 30.3 SFA diet: BMI 31.3	61 (M/F)	30–65	10 weeks parallel	PUFA diet: (received foods rich in n6 linoleic acid, ie,scones (baked-on sunflower oil), margarine, sunflower oil, and sunflower seeds) corresponding to 15% of energy as linoleic acid. SFA diet: (received scones (baked-on butter) and butter). No altering their intakes of total fat and the type and amount of carbohydrates and protein.	↔		↔			
Telle-Hansen et al., 2012, Norway	Healthy BMI 27–40	23 (M/F)	18–70	12 weeks parallel	Test products: 20 g margarine, 11 g mayonnaise (both rapeseed oil based), 12 g flaxseed oil. High ALA-DAG: 8.29 g/day. High ALA-TAG: 8.51 g/day.	↔					
^a^Van Dijk et al., 2012, The Nederlands	Lean, obese, or obese with diabetes	42 (M)	50–70	0–4 h post-prandial cross-over	Isocaloric milkshake with 95 g of fat (88% of energy) SFA shake: 95 g palm oil (54% of energy/total fat) MUFA shake: 95 g high oleic sunflower oil (83% of energy/total fat) n-3 PUFA shake: 40 g palm oil and 55 g Marinol D-40 (40% DHA) (40% of energy/total fat)		↔				↑mRNA with MUFA and n3 PUFA vs. SFA
Esser et al., 2013, The Netherlands	Lean BMI 23.8 Obese BMI 32.4	18 lean 18 obese (M/F)	61.8 / 62.6	0–4 h post-prandial Cross-over	Isocaloric milkshake with 95 g of fat (88% of energy) SFA shake: 95 g palm oil (54% of energy/total fat) MUFA shake: 95 g high oleic sunflower oil (83% of energy/total fat) n-3 PUFA shake: 40 g palm oil and 55 g Marinol D-40 (40% DHA) (40% of energy/total fat)	↔		↔	↔	↔	
Lee et al., 2014, USA	Early stage T2DM or MetS BMI > 23 and < 45	59 (M/F)	> 21	8 weeks parallel	Corn oil (CO): 9 daily CO capsules (3.9 g LA and < <0.01 g EPA pr day. Botanical oil (BO): combination of echium and borage oils); 10 daily BO capsules (7 echium and 3 borage) (1.80 g LA and 0 g EPA pr day). Fish oil (FO): 9 daily FO capsules. (0.48 g LA and 3.58 g EPA pr day)	↔					
Rozati et al., 2015, USA	Healthy BMI 29	41 (M/F)	72	12 weeks parallel	Olive oil/spread group (OO): extra virgin olive oil Control oil/spread group (CON): 10% corn oil, 90% soybean oil, spread based on butter		↔	↔			
Stonehouse et al., 2015, Australia	Healthy BMI 25–35	28 (M)	32–65	1–5 h post-prandial Cross-over	Chicken fried in oil, bread fried in oil and a small salad PO: 40 g palmolein oil OO: 40 g olive oil					↔	
Teng et al., 2015, Malaysia	MetS BMI 30.9	30 (M/F)	33.8	0–6 h post-prandial cross-over	CARB meal: high carbohydrate/low fat MUFA meal: monounsaturated fatty acids-enriched PUFA meal: polyunsaturated fatty acids-enriched SFA meal: saturated fatty acids-enriched	↔	↔	↔			
Mixed diets											
Camhi et al., 2010, USA, The Diet and Exercise for Elevated Risk Trial (DEER)	Dyslipidemic and MetS BMI >32	278 (M/F)	F: 57.6 M: 49	1 year parallel	Control group: no intervention Exercise group: aerobic exercise only Diet group: NCEP step 2 diet Diet-plus-exercise group: both aerobic exercise and NCEP step 2 diet	↓with diet and diet + ex (in women with MetS)					
De Mello et al., 2011, Finland, The Sysdimet study	Impaired glucose metabolism BMI 31.1	104 (M/F)	59	12 weeks parallel	Healthy diet: wholegrain cereals, wholemeal pasta, fatty fish, vegetable oil and vegetable oil products, bilberries Whole-grain-enriched diet (WGED): wholegrain cereals, wholemeal pasta, one whole grain oat snack bar per day, not to change their current fish and berry consumption. Control group: avoid wholegrain cereals and pasta, no bilberry intake, fatty fish only 1 a week	↓with WEGD vs. control (without statin users)	↔	↔			
Uusitupa et al., 2013, Nordic multicenter study, The SYSDIET study	MetS BMI 31.6	166 (M/F)	55	18–24 weeks parallel	Healthy Nordic diet: ≥ 25% of total energy as whole grain of which ≥ 50%as rye, barley or oat. Whole-grain pasta and unpolished rice (≥ 6 g fiber/100 g) ≥ 2–3 meals per week. Low salt content (≤ 1%) in breads cereals with no added sugar or honey. Bread (≥ 6 g fiber/100 g) ≥ 6 slices per day. Fruits, vegetables and berries ≥ 500 g/day, rapeseed oil, low fat liquid dairy ≤ 1% of fat, cheese ≤ 17%, fatty fish, white meat, fruit and berry juices. Control diet: refined cereals and wheat. 200–250 g fruits and vegetables, no bilberries, butter, no limits of dairy products, ≤ 1 meal of fish per week, meat/beverages: no limitation	↔		↔			
Lesna et al., 2013, Czech Republic	Dyslipidemic BMI 31.63	14 (F)	>45	3 weeks cross-over	SAFA diet: a fully controlled diet high in SFA PUFA diet: a fully controlled diet high in PUFA	↓with PUFA vs. SFA diet					
The LIPGENE study											
Petersson et al., 2010, Pan European, The LIPGENE study	MetS BMI 20–40	417 (M/F)	35–70	12 weeks parallel	HSFA: high SFA HMUFA: high MUFA LFHCC-n3: low fat high complex carbohydrate w/ 1.24 g/day LC omega-3 PUFA LFHCC (placebo): low fat high complex carbohydrate w/ 1 g/day high oleic sunflower oil	↔					
Perez-Martinez et al., 2010, Pan European, The LIPGENE study	Met BMI 20–40	74 (M/F)	35–70	0–8 h post-prandial challenge (after 12 weeks intervention) parallel	HSFA: high SFA HMUFA: high MUFA LFHCC-n3: low fat high complex carbohydrate w/ 1.24 g/day LC omega-3 PUFA LFHCC (placebo): low fat high complex carbohydrate w/ 1 g/day high oleic Sunflower oil				↓with HMUFA vs. HSFA and LCHCC n3	↔	
^a^Meneses et al., 2011, Pan European, The LIPGENE study	MetS BMI 20–40	39 (M/F)	35–70	0–4 h post-prandial challenge (after 12 weeks intervention) parallel	HSFA: high SFA HMUFA: high MUFA LFHCC-n3: low fat high complex carbohydrate w/ 1.24 g/day long chain omega-3 LFHCC (placebo): low fat high complex carbohydrate w/ 1 g/day high oleic Sunflower oil			↔ ↔mRNA			↔ ↔mRNA
Tierney et al., 2011, Pan-European, The LIPGENE study	MetS BMI 20–40	417 (M/F)	35–70	12 weeks parallel	HSFA: high SFA HMUFA: high MUFA LFHCC-n3: low fat high complex carbohydrate w/ 1.24 g/day LC omega-3 PUFA LFHCC (placebo): low fat high complex carbohydrate w/ 1 g/day high oleic Sunflower oil	↔	↔	↔	↔	↔	
^a^Cruz-Teno et al., 2012, Pan European, The LIPGENE Study	MetS BMI 20–40	75 (M/F)	35–70	0–4 h post-prandial challenge (after 12 weeks intervention) parallel	HSFA: high SFA HMUFA: high MUFA LFHCC-n3: low fat high complex carbohydrate w/ 1.24 g/day LC omega-3 PUFA LFHCC (placebo): low fat high complex carbohydrate w/ 1 g/day high oleic Sunflower oil		↔ ↓mRNA with HMUFA vs. HSFA diet				↓with HMUFA and LCHCC n3 vs. HSFA
Camargo et al., 2013, Pan European, The LIPGENE Study	MetS BMI 20–40	39 (M/F)	35–75	0–4 h post-prandial challenge (after 12 weeks intervention) parallel	HSFA: high SFA HMUFA: high MUFA LFHCC-n3: low fat high complex carbohydrate w/ 1.24 g/day LC omega-3 PUFA LFHC (placebo): low fat high complex carbohydrate w/ 1 g/day high oleic Sunflower oil	*No between groups measures were performed*					
Rangel-Zuniga et al., 2015, Pan European, The LIPGENE Study	MetS BMI 20–40	417 (M/F)	35–70	12 weeks parallel	HSFA: high SFA HMUFA: high MUFA LFHCC-n3: low fat high complex carbohydrate w/ 1.24 g/day LC omega-3 PUFA LFHCC (placebo): low fat high complex carbohydrate w/ 1 g/day high oleic Sunflower oil	*No between groups measures were performed*					

^a^Studies including results at both protein and gene level. Results at gene level are indicated (mRNA)

The symbols reflect statistical significant increase (↑) or decrease (↓) between groups, or no change (↔) between groups

**Table 2:** Weight reduction studies and inflammatory response

Study	Subjects	*N* (sex)	Age (years)	Duration and study design	Intervention/intake	CRP	TNFα	IL-6	ICAM	VCAM	MCP1
Bazzano et al., 2014, USA	Healthy BMI 30–45	103 (M/F)	22–75	12 weeks parallel	Low-fat diet: less than 30% of daily energy intake from total fat (with < 7% from SFA) and 55% from carbohydrates Low-carbohydrate diet: less than 40 g/day of digestible carbohydrates (total carbohydrates—fiber)	↓with low-carbohydrate vs. low-fat					
De Luis et al., 2014, Spain	Healthy BMI 36.5	391 (M/F)	438	12 weeks parallel	Diet P: high polyunsaturated fat hypocaloric diet (34.4 E%) Diet M: high monounsaturated fat hypocaloric diet (34.1 E%)	↔					
Silver et al., 2014, USA	Healthy BMI 34.8	91 (F)	36.7	14 weeks parallel	Testing 18C fatty acid supplementation Balanced high fat diet (HFD) supplemented with 9 g/day stearate, oleate, linoleate, or placebo (food grade silicon dioxide powder)	↔	↑with HFD + O vs. HFD + P	↑with HFD + O vs. HFD + P			
Su et al., 2015, Taiwan	MetS BMI ≥ 24 and ≤ 35	143 (F)	–	12 weeks parallel	CR: calorie restriction diet CRMR: calorie-restriction meal-replacement diet CRF: Calorie-restriction diet with fish oil supplement CRMRF: calorie-restriction meal-replacement diet with fish oil supplementation	↔		↔			
Tovar et al., 2016, Sweden	Healthy BMI 25–33	47 (M/F)	50–73	8 weeks parallel	Multifunctional diet (MFD): low-glycemic-impact meals, antioxidant-rich foods, oily fish, viscous dietary fibers, soybean and whole barley, kernel products, almonds and plant stanols Control diet (CD): processed cereals, white wheat bread, dark wheat bread, fruits and vegetables	↔					

The symbols reflect statistical significant increase (↑) or decrease (↓) between groups, or no change (↔) between groups

#### Dairy products

Dairy products include a vast amount of different products, consisting of many different nutrients, bioactive compounds and bacteria. During the last years, several studies have been conducted to improve our knowledge of the possible health effects of milk and dairy products. Drouin-Chartier and coworkers investigated the effect of intake of milk (3.2 servings of 2% fat milk/day) compared with no servings of milk/day in a randomized cross-over study where each period lasted for 6 weeks [20]. No difference was seen in levels of CRP, VCAM, ICAM, and E-selectin after the treatment in any of the groups (Table 1). The authors concluded that short-term milk intake has no observable favorable or deleterious effects on cardio-metabolic risk factors [20]. Furthermore, fermented dairy products have been shown to have cardio-protective effects [21]. Using a cross-over design, Nestel et al. investigated the effects of full-fat fermented (cheese and yogurt), full-fat non-fermented (butter and cream) and low-fat (milk and yogurt) dairy products in obese subjects [22]. Each of the three interventions lasted for 3 weeks. The low-fat products had half the amount of total fat and a quarter amount of SFA than the full-fat products, while MUFA and PUFA content were similar in all groups. They did not find any changes in the inflammatory markers CRP, TNFα, ICAM, VCAM, IL-1β, and MIP-1a except for increased levels of IL-6 in the full-fat non-fermented group, compared to the other groups. Moreover, Van Meijl and Mensink investigated the effects of low-fat dairy products on low-grade systemic inflammation and endothelial function in overweight and obese subjects [23, 24]. Subjects were randomly allocated to consume 500 mL low-fat (1.0%) milk and 150 g low-fat (1.5%) yogurt or 600 mL of fruit juice and 43 g fruit biscuits (corresponding to three biscuits). Each study period was 8 weeks. Daily intake of dairy products compared to fruit juice did not change fasting plasma concentration of CRP [24] or TNFα, IL-6, ICAM, VCAM, MCP1, or sTNFR1 [23]. However, sTNFR2 was increased after dairy compared to control food intake [23] (Table 1).

Considerable public interest has been focused on minimally processed products, mainly because they are believed to contain more natural ingredients, nutrients and bioactive molecules, and thereby appear healthier. In this respect, *trans* fatty acids occur naturally in fats from ruminants, and fats produced using certain industrial processes, and are known to have negative cardio-metabolic effects. However, CLA, a natural *trans* fatty acid, has been examined for possible beneficial health effects in several studies [25, 26]. Venkatramanan et al. investigated the effect of milk enriched with natural CLA or enriched with synthetic CLA, compared to untreated milk, in a randomized three-phase cross-over single-blind trial [27]. Each dietary phase lasted for 8 weeks. The amount of total fat was similar in the study products (approx. 4.0%) but the level and type of CLA differed; however, there was no difference in any of the measured inflammatory markers between the three dietary groups [27]. In another study, they compared buns with butter made of milk from grazing versus conventional fed cows [28]. The fatty acid profile in both the butter and buns were similar. After 12 weeks of intervention, there were no differences in inflammatory markers between the groups [23] (Table 1).

The six studies included in this review compare quite different dairy products. Two of the studies found minor differences in inflammatory markers. Whereas the full-fat and non-fermented products slightly increased inflammation, only a limited amount of inflammatory markers changed in each study. Therefore, from the studies included in the present review, it seems that intake of dairy products have no favorable or deleterious effects on inflammation (CRP, TNFα, ICAM, VCAM, IL-1β, sTNFR1, and MIP-1α) in overweight and obese subjects. Nevertheless, dairy products may have beneficial effects by lowering CRP levels in obese subjects [8] not captured in studies included in the present review, and the effect may be related to non-lipid content of the dairy products, such as matrix effects or dairy peptides. In the present review, the focus was fat intake, and therefore no studies specifically investigating matrix effects or dairy peptides were included, which may explain the lack of beneficial effects.

#### Nuts

Nuts are high-energy, nutrient-dense foods that are rich in PUFA and other bioactive components, including fiber, antioxidants, vitamins, and minerals [29]. Evidence suggests that nut consumption may have beneficial effects on oxidative stress, inflammation, and vascular reactivity [29]. Importantly, epidemiological studies show a negative correlation between nut intake and risk of CVD [29] and recently, the PREDIMED study found 30% reduction in CVD after intake of a Mediterranean diet enriched with mixed nuts (walnuts, almonds, and hazelnuts) in a high-risk CVD group [30].

Five studies that investigated the inflammatory effects of nuts in overweight and obese subjects were included in this review (Table 1). When investigating the effect of the American Heart Association (AHA) dietary guidelines with or without enrichment of 30 g raw mixed nuts (15 g walnuts, 7.5 g almonds, and 7.5 g hazelnuts) on inflammatory markers for 12 weeks, Lopez-Uriarte et al. did not find any changes in VCAM and ICAM between the groups [31]. However, VCAM was reduced within the nut group. In the same study, Casas-Agustench and coworkers investigated the effect on CRP, MCP1, IL-18, and IL-6 [32]. A moderate weight loss was observed in both groups. MCP1 and IL-18 levels decreased after both diets, with no differences between the groups. The level of IL-6 was reduced in the nut group only; however, the significance disappeared after adjusting for weight loss. Interestingly, no differences were seen for plasma levels of CRP, neither within nor between the groups [32]. Furthermore, Bakhtiary et al. did a 12-week randomized controlled study among elderly women with metabolic syndrome (MetS). Studying the effect of intake of soy nuts or textured soy protein, they found no significant differences in CRP between the groups [33]. In another study, the effects of high oleic peanuts on cardio-metabolic measures and CRP in healthy, overweight adults were examined; however, no clear effects were found [34]. Finally, Tey and coworkers compared the effects on inflammatory markers with consumption of either 0, 30, or 60 g of hazelnuts per day for 12 weeks [35]. They found no effect on any of the inflammatory markers examined (CRP, IL-6, ICAM, and VCAM).

Despite the high-fat content of nuts, including PUFA, intake of nuts do not seem to modulate markers of inflammation in overweight or obese individuals, which is in accordance with previous review [8]. Even though two of the five included studies found lower inflammatory markers in the nut groups (IL-6, VCAM), the effects disappeared when adjusting for weight loss or compared to the control group.

#### Vegetable oils

Vegetable oils are rich in PUFA, the main constituent being n6 fatty acids. Even though n6 fatty acids are widely considered pro-inflammatory while n3 fatty acids are considered anti-inflammatory, the evidence supporting the former is contradictory and inconclusive [6]. In addition, there is strong evidence that n6 fatty acids promote health by reducing LDL cholesterol and thereby the risk of CVD [30, 36–40]. Our group previously showed no change in CRP among overweight and obese subjects after 12 weeks of intervention with food items where alpha-linolenic acid rich triglyceride oil was substituted with alpha-linolenic acid rich diacylglycerol oil [41] (Table 1). In a randomized, parallel single-blind study, Gagliardi and coworkers investigated the effects of daily servings of butter, no-*trans*-fat margarine or plant sterol margarine on biomarkers of inflammation and endothelial dysfunction [42]. No significant difference between the groups was found on the concentration of inflammatory and endothelial dysfunction markers. Moreover, Bjermo et al. investigated the effects of a high-PUFA diet (vegetable oil) or high-SFA diet (mainly butter) on liver fat, systemic inflammation, and metabolic disorders in a randomized study lasting for 10 weeks (the HEPFAT study) [43]. Compared to the SFA group, liver fat, IL1Ra, and sTNFR2 were lower in the PUFA group at the end of the study. In contrast, no group difference was found for plasma levels of CRP, IL-1β, IL-6, and IL-10 [43]. Moreover, in an 8-week randomized single-blind parallel intervention study, Lee and coworkers compared the effect of three PUFA-based supplements, corn oil, a botanical oil, or fish oil. None of the supplements changed the level of CRP within or between groups [44]. In an 8-week single-blind, randomized trial, Rozati et al. investigated the effects of extra virgin olive oil, compared with corn oil, soybean oil, and butter, on quantity and functionality of a number of lymphocyte subsets [45]. In general, there were no anti-inflammatory effects of olive oil intake; however, the authors suggested that the increased T cell proliferation in the olive oil group might reflect immune-modulatory effects of olive oil consumption [45].

Masson and Mensink [46], Esser and coworkers [47], and van Dijk et al. [48] all studied the difference in post-prandial response after intake of SFA or PUFA in overweight subjects. Masson and Mensink compared a butter meal (14 E% SFA) and a margarine meal (8.7 E% PUFA of which 8.0 E% was linoleic acid). Compared with the SFA meal, the PUFA meal decreased IL-6, TNFα, sTNFR1 and sTNFR2, and sVCAM [46]. Esser et al. and Van Dijk et al. reported data from the same study, where three different high-fat milkshake meals consisting of palm oil, high oleic sunflower oil, or n3 PUFA (DHA) [SFA-meal (51 E% SFA), MUFA-meal (79 E% MUFA) and PUFA-meal (38 E% PUFA)] were compared in both obese and lean subjects [47, 48]. Subjects with MetS [47] and lean, obese, and obese-diabetic subjects [48] were included in the studies. In the work by Esser et al., all groups displayed a post-prandial increase in many of the measured inflammatory markers, including IL-8, sICAM-3, sICAM-1, sVCAM-1, and a decrease in IL-6. In contrast, and despite clear differences in post-prandial triglyceride response and higher baseline values of CRP in obese subjects, they found no global differences between the groups on inflammatory response [47]. Still, SFA consumption was associated with higher plasma P-selectin concentration 2 h post-prandial compared with MUFA and n3 PUFA, and lymphocyte CD11a and CD11b expression decreased in lean participants but did not change in obese subjects [47]. Van Dijk et al. found that plasma concentrations of IL-1β varied both according to the type of fat and groups [48]. In addition, the TNFα level was lower after the intervention in lean compared with obese and obese diabetic subjects [48]. Palmolein, derived from dry fractionation of palm oil, is a rich source of both SFA (42%) and MUFA (47%), in addition to some PUFA (12%), and is widely used for both frying and replacement of *trans* fat. In a post-prandial study with isocaloric high protein, high-fat meals prepared with either palmolein or olive oil, Stonehouse and coworkers observed no differences on endothelial function 1–5 h after intake of a high protein meal consisting of 40 g of either olive oil or palmolein in overweight and obese men [49]. Finally, in another post-prandial, cross-over study in 30 obese males and females, investigating the inflammatory effects of a high-carbohydrate diet, high-MUFA diet (high oleic sunflower oil), high-PUFA diet (sunflower oil), or a high-SFA diet (palm oil), the researchers found no differences between groups with regards to CRP, TNFα, and IL-6 [50].

Although tissue biopsies are often limited in human studies, blood samples are easily accessible. Peripheral blood mononuclear cells (PBMC) are a subset of the white blood cells that include monocytes and lymphocytes. The PBMC, as part of the immune system, are exposed to many of the same environmental factors as metabolic tissues and provide a model for human metabolic regulation and inflammation on, for example, gene expression level [51, 52]. In addition to the circulating inflammatory markers, Van Dijk et al. also measured the effect of high SFA (palm oil), high MUFA (high oleic sunflower oil), and high n3 PUFA (DHA) on gene expression in PBMC [48]. Intake of high-fat MUFA and n3 PUFA shakes compared to SFA shakes, induced higher increase in the expression of MCP1 and IL-8 mRNA levels in PBMC [48] (Table 1).

In the present review, 3 of 10 studies with vegetable oils found some beneficial effects on inflammation [43, 47]. Except for some contradictory effects observed at gene expression level in the study by Van Dijk et al. [48], none of the studies found convincing evidence of increasing circulating inflammatory markers after intake of vegetable oils. Hence, vegetable oils do not seem to have detrimental inflammatory effects in overweight and obese subjects, and might even have some beneficial effects. These findings correlate with different studies showing reduced risk of CVD and cholesterol after intake of n6 fatty acids. Ulven et al. obtained 11% reduction in LDL cholesterol when replacing 5% of the energy from SFA with PUFA, mainly linoleic acid, with no effect on inflammatory markers in normal weight individuals [38]. Farvid and coworkers have shown that by replacing 5% of the energy from SFA with linoleic acid, gives a 13% reduction in risk of coronary artery disease (CAD) mortality and 9% in cardiac events [37]. In the PREDIMED study, a 13% reduction in risk of stroke, heart infarction and cardiovascular mortality was obtained with a diet high in linoleic acid [30]. In addition, epidemiological studies show similar results [39].

#### Mixed diets

Humans eat mixed diets, not single nutrients. Consequently, researchers have shifted focus to examine the health effects of complex diets and dietary patterns instead of the classic reductionist approach. The Mediterranean diet has long been related to improved health, while a westernized diet has the opposite effect. Similarly, a healthy Nordic diet associate with lower mortality and improve cardiovascular risk factors [53, 54]. Both Uusitupa and De Mello have investigated the effects of a healthy Nordic diet on inflammatory markers in obese individuals with MetS [55] or impaired glucose metabolism [56], respectively. In those studies, a healthy Nordic diet included whole grain (of which ≥ 50% rye, barley, and oat), cereals, fruits, vegetables and berries, rapeseed oil, margarine, low-fat dairy products, fish, white meat, and avoidance of sugar-sweetened beverages. The control diet included refined cereal products (of which ≥ 90% as wheat) and butter, less fruits and vegetables and no berries, and less than one meal of fish per week. There were no limits of dairy products, meat and sugar-sweetened beverages. Uusitupa et al. found decreased levels of IL1Ra with the healthy diet compared with the control diet. In addition, there was an association between intake of saturated fats and IL1Ra [55] (Table 1). De Mello et al. found decreased levels of E-selectin in the healthy diet group compared with the control diet group, and CRP levels decreased both within the healthy diet and whole grain enriched diet (WGED), but there were no difference when compared with control diet [56]. Furthermore, Camhi et al. examined CRP changes in subjects with MetS enrolled in a lifestyle intervention trial with factorial design (the DEER trial) [57, 58]. Subjects were randomly assigned to a control group, an exercise group, a diet group (NCEP-II guidelines), or a diet and exercise group. CRP was reduced in the two latter groups, but in women only. In men and in women and men combined, however, CRP was unchanged. In addition, in that study, there was no effect of exercise on CRP levels [57]. Finally, in a fully controlled study with 14 overweight and obese, dyslipidemic women, CRP levels decreased after 3 weeks with a high-PUFA diet compared to a high-SFA diet [59] (Table 1).

Measuring the effects of single nutrients or foods might be difficult due to sensitivity of the analyses, as well as the fact that many nutrients are correlated in dietary patterns. In the present review, three of the four studies decreased levels of CRP or other inflammatory markers after intervention with mixed diets, in addition to some within group effects. The NCEP-based diet, a Nordic diet, and a high-PUFA diet all showed beneficial effects on inflammation, as evidenced by decreased CRP, E-selectin, and IL1Ra. This finding indicates that a change in whole diet is more effective with regard to inflammation compared to change of single components of the diet. This may be explained by the accumulation of many small effects and the synergy of single nutrients, or that compliance is easier for participants when given a whole diet instead of single nutrients or foods, thereby also contributing to decreasing confounding factors and practical obstacles. Several studies have shown beneficial health effects of exchanging SFA with PUFA, and in the review by Calder et al., they suggest that Mediterranean diet, characterized by high intake of PUFA, may lead to decreased chronic low-grade inflammation [8]. Hence, it is conceivable that a healthy fatty acid composition (high PUFA and low SFA) as part of a healthy dietary pattern may be of importance in reducing systemic low-grade inflammation when overweight or obese.

### The LIPGENE study

In the pan-European LIPGENE study, researchers investigated whether high-SFA diet (HSFA), high-MUFA diet (HMUFA), or low-fat, high-complex carbohydrate diets supplemented with either sunflower oil (LFHCC-SFO) or long chain (LC) n3 fatty acids (LFHCC-LC n3) for 12 weeks affected CRP [60], IL-6, TNFα, ICAM, or VCAM [61] in subjects with MetS (Table 1). No diet-specific effects were found. In contrast, after 12 weeks of intervention, a post-prandial fat challenge was performed, and ICAM [62] was reduced with the HMUFA diet compared with the LFHCC-LC n3 and HSFA diets, while MCP1 [63] were reduced with both HMUFA and LFHCC-LC n3 diets compared to HSFA diet. In contrast, in later analyses, Meneses and coworkers did not find any changes in IL-6 or MCP1 after the post-prandial fat challenge [64].

Dietary fatty acids may affect health via several mechanisms, for example by influencing the activity of transcription factors involved in metabolic regulation and inflammation, like NF-κB and PPARγ [6, 8]. Changes in adipose tissue gene expression was studied in the LIPGENE study, and it was shown that the mRNA level of p65 (sub-unit of the NF-κB transcription factor) was induced by a HSFA challenge meal, but with no apparent difference between the four different test meals [64]. Post hoc statistical analyses, however, showed that the p65 gene expression level was increased after intake of LFHCC-SFO and LFHCC-LC n3 diets. In addition, the post-prandial (4 h) IκBa gene expression in PBMC was increased after HSFA compared with LFHCC-LC n3 diets [63]. In contrast, fasting IκBa gene expression level was increased after 12 weeks of intervention and post-prandial (0 h) with the LFHCC-LC n3 diet compared with HSFA and MUFA diets. Furthermore, post-prandial PBMC TNFα and MMP-9 mRNA levels were reduced after intake of the HMUFA compared with the HSFA diet. After the HMUFA post-prandial fat challenge, the mRNA level of TNFα and metalloproteinase-9 (MMP-9) were downregulated in PBMC compared with the HSFA fat challenge [63]. Moreover, the post-prandial effect on the PBMC proteome (nuclear and cytoplasmic) was also examined in the LIPGENE study by Rangel-Zuniga et al. [65] and Camargo et al. [66]. The PBMC proteome (nuclear and cytoplasmic) displayed changes in pro-inflammatory proteins after intake of HMUFA (F2, TLN1, GSN, CAPZ) and LFHCC-LC n3 diets (FGB, FGG, VCL, ACTB, CAPZA1, and MACF1) [65]. Pathways analysis showed that inflammatory response protein was differently expressed 4 h after intake of four different meals with different fat quality. In particular, pathway analysis showed that the top function associated with protein differently expressed after intake of a HSFA meal was inflammatory response (HLA-C, THBS1, and PSME1 were upregulated, and PLEC, FGB, and HSPA1A were downregulated) [66] (Table 1).

Inflammatory markers, both circulating and mRNA levels (TNFα, ICAM, MCP1, p65, and MMP-9), were investigated in the LIPGENE study, in which four of the seven studies investigated gene expression and or proteome effects. Taken together, the results from the LIPGENE study suggest that a high-MUFA diet, and to a lesser extent a LFHCC diet, have some anti-inflammatory effects compared with high-SFA diet. However, only two of the studies showed effects on circulating inflammatory markers, both investigating effect after a fat challenge; the other studies showed no effect, and it is therefore difficult to draw firm conclusions.

#### Fatty acids in weight reduction studies

Diet-induced weight reduction effectively improves obesity-related metabolic aberrations and low-grade inflammation. However, the importance of dietary fat in the rising prevalence of overweight and obesity is unknown. While some studies show no association between dietary fat intake and body weight, other studies do find an association [67, 68]. However, during the past years, several studies have confirmed that total energy intake, rather than macronutrients distribution per se, is the more important determinant of weight reduction and maintenance [69, 70]. Therefore, it is of high interest to elucidate if different fatty acids, as part of a diet-induced weight reduction, will affect the low-grade inflammatory status observed in overweight and obesity.

Tovar and coworkers [71] investigated the cardio-metabolic protective effect of an 8-week multifunctional diet (MFD; including food items such as low-glycemic-impact meals, antioxidant-rich foods, oily fish, viscous dietary fibers, soybean and whole barley, kernel products, almonds, and plant stanols) in overweight and obese subjects, compared with a control diet where none of the abovementioned functional food items were included. Some of the food items were included in the control diet was provided such as white wheat bread, dark wheat bread, parboiled rice, cornflakes, champignon spread, prawn spread, powdered pastry cream, fruit preserves, and mango chutney. Both diets were designed according to the Nordic nutrition recommendations. Both diets resulted in a weight loss of approximately 2%. There was no difference between the diets in levels of CRP between the groups (Table 2). Silver et al. tested the effect of a 16-week, high-fat diet-based weight reduction intervention [72]. The high-fat diets were supplemented with either 9 g/d of stearate (18:0), oleate (18:1), linoleate (18:2), or placebo, and a panel of inflammatory markers was measured. All four groups each lost 5 kg weight, which accounted for most of the observed effects on the inflammatory markers (IL-1a, IL-1β, IL-12, IL-17, IFNγ, TNFα, and TNFβ). Using linear mixed models, adjusting for weight change and compared with the control group, the authors showed that the main effects of 18:0 was a drastic reduction of IFNγ, and the main effects of 18:1 was a small increase in IL-1β, IL-6, IL-10, IL-12, and TNFα. Surprisingly, there were no main effects of 18:2 [72]. In a 12-week weight reduction trial, De Luis et al. investigated the effects of a PUFA or MUFA diet, as well as interaction with GLP-1 variants, on CRP [73]. They found no differences in CRP with either the dietary interventions or the genetic variants. Su et al. investigated the effect of n3 supplementation on inflammatory markers in the context of a 12-week weight reduction [74]. Women with MetS were randomly assigned to one of four interventions: energy restriction, energy restriction with meal replacement, energy restriction with fish oil, or energy restriction with meal replacement and fish oil. Although they found a small additive effect of n3 supplementation, the authors concluded that the weight reduction was a more important determinant of changes in inflammatory markers than fish oil intake [74]. Moreover, the effect of low-fat versus low-carbohydrate diets on inflammation was investigated in 103 males and females for 12 weeks [75]. The low-fat diet group ingested less than 30 E% from fat (with < 7 E% from SFA) and 55 E% from carbohydrates, while the low-carbohydrate diet group had less than 40 g per day of digestible carbohydrates (total carbohydrates minus fiber). Compared to the low-fat diet, intervention with the low-carbohydrate diet decreased CRP levels [75] (Table 2).

Weight reduction per se will have positive cardio-metabolic effects, including reduced inflammation [76]. However, only one of the weight reduction studies included in this review found a beneficial effect on CRP between groups with different dietary fat composition. Moreover, one of the studies found that a high fat diet supplemented with oleate compared to placebo increased several inflammatory markers despite a weight reduction [72]. The present review do not focus on weight reduction and inflammatory response, but rather fat quality and inflammatory response, which might explain the discrepancy between the present review compared with previous studies [76]. The limitation in time, giving a limited amount of studies in the present review may also influence the results.

## Conclusion

Obesity is associated with pathological changes in adipose tissue morphology, including infiltration of immune cells, and obese individuals have higher circulating levels of inflammatory markers than lean individuals [5, 7]. Associations between intake or status of various fatty acids and inflammatory markers have been examined in human studies and there is a general agreement that increasing dietary SFA intake, especially in overweight and obese individuals, is associated with raised inflammatory markers [8]. In the present review comprising studies between January 2010 and September 2016, we only found minor effects of dietary fat on inflammatory markers in overweight and obese subjects. The most consistent effects were found after intervention with whole diets. Dairy products, vegetable oils and fatty acids in dietary weight reduction studies only showed minor effects, while nuts did not seem to have any effects on inflammatory markers. Due to small effects, large inter-individual differences and insensitive methods, dietary health effects are difficult to measure. This might explain why we do not find any effects after intervention with single nutrients or foods in the present review. However, nutrients in whole diets may have synergistic effects, and thereby be able to affect the inflammatory system with more beneficial effects. To measure diet-induced changes, it may be necessary to temporarily disrupt the body’s homeostasis, which may be done with dietary challenge tests. The extent of the disruption and the speed of recovery to homeostasis may be considered as health indicators [77].

Only two of the studies investigating the effect of mixed diets found differences between subgroups among the intervention groups. In the study by De Mello et al., the CRP level was significantly reduced after the WEGD diet compared with the control diet, but only when excluding statin users [56]. Similar, CRP level was reduced between the diet- and diet/exercise group compared with the control group, but only in women with MetS [57]. Due to the small effect sizes expected from dietary interventions, medications that influence inflammation may camouflage a real effect. In addition, different effect sizes between men and women are commonly registered. This is maybe due to physiological or pathophysiological differences between the sexes, or differences in compliance and motivation. Finally, subtle differences in baseline level of inflammation may appear as differences in response to diet. Responses to dietary challenges post-prandial may be more informative than measurement of fasting homeostatic measures. In the present review, nine articles included post-prandial measures, in which five found changes in inflammatory response. Hence, a dietary challenge apparently showed a more consistent response. However, four articles were from the LIPGENE study, therefore making it difficult to conclude if studies including a challenge are more efficient in differentiating the response elicited by diet.

Investigating the transcriptomic profile in different cells can improve our understanding of the metabolic regulation and provide insights into the mechanisms of metabolic disease. Investigating metabolic responses of food and nutrients optimally involve tissue biopsies. However, invasive biopsy procedures are a limiting factor in human studies, while blood samples are easily accessible. Diet-induced changes in concentration of circulating inflammatory markers compared to tissue concentrations may be undetectable, but the PBMC gene expression measurements may be more sensitive than plasma proteins to alterations in circulating nutrients. Also, PBMC are hypothesized to be a relevant substitute for investigating metabolic regulation in tissues and are frequently used as an indirect measure, particularly of gene regulation [51, 52]. Svahn et al. compared the transcriptome effect of dietary fat in different organs. In contrast to the hypothesis stated above, they found that dietary fatty acids affected the transcriptome in distinct manners in different organs [78], demonstrating the complexity of diet-gene interactions and the interpretation of dietary intervention studies. The present review did not find obvious effects of dietary fat on gene expression or proteomic related to inflammation; however, few studies investigating gene expression or proteome were included.

Obese individuals represent a heterogeneous group with different phenotypes. Interestingly, a subgroup of obese individuals has been described as “metabolically healthy obese” (MHO). In contrast to at-risk obese (ARO), the MHO phenotype is defined by a favorable cardio-metabolic profile, despite the same amount of body fat, including a more favorable inflammatory profile, less visceral fat, less infiltration of macrophages into adipose tissue, and smaller adipocyte cell size [52, 79–81]. The studies included in the present review have not presented data of differentiated effects in different obese phenotypes. It has been estimated that 18–44% of all obese may be categorized as MHO [82] and because the effect sizes may be small, it may be difficult to detect dietary effects on inflammation when investigating all obese as one group. During the past years, increasing focus has been given to the relation between gut microbiota and health. Several studies have confirmed a direct implication of gut microbiota in obesity progression [83] and gut microbiota is established as a determining factor in obesity-related inflammation [83]. Gut microbiota are affected by several factors, including dietary factors like fat quality and quantity and fiber. The present review investigates inflammatory-modulating effects of mainly dietary fat quality, and to some degree quantity. However, the proportion of fat versus other dietary components, like fiber or the composition of gut microbiota, is not included in any of the studies.

In the present article, only a restricted part of the scientific field has been reviewed. Our literature search was limited to randomized controlled trials with fatty acids in overweight and obese subjects, published between 2010 and 2016 that included measurement of inflammatory markers. In addition, we have categorized the included studies according to type of dietary intervention. Hence, some of the studies may be relevant in more than one category, which may have affected the conclusion. Taken together, in the present review we find minor changes in inflammation after modulating fat intake in overweight and obese subjects. Even though randomized controlled trials are superior when studying cause-and-effect, they do not necessarily have a mechanistic approach. To progress our understanding on how diet and dietary components affect our health, mechanistic studies are required. Hence, future studies should include whole diets and characterization of obese phenotypes at a molecular level, including omics data and gut microbiota to help us understand the role of diet on low-grade inflammation in overweight and obese subjects.

## Abbreviations

ARO: At risk obese
BMI: Body mass index
CAD: Coronary artery disease
CRP: C-reactive protein
CVD: Cardiovascular disease
E%: Energy %
ICAM: Intercellular adhesion molecule
MCP-1: Monocyte chemoattractant protein-1
MetS: Metabolic syndrome
MHO: Metabolically healthy obese
MMP-9: Matrix metalloproteinase-9
MUFA: Monounsaturated fatty acid
n3/n6: Omega-3/omega-6
PBMC: Peripheral blood mononuclear cells
PUFA: Polyunsaturated fatty acid
RCT: Randomized controlled trial
SFA: Saturated fatty acid
T2D: Type 2 diabetes
TNFa: Tumor necrosis factor alpha
VCAM: Vascular cell adhesion protein
